# Personality traits and complex problem solving: Personality disorders and their effects on complex problem-solving ability

**DOI:** 10.3389/fpsyg.2022.788402

**Published:** 2022-08-03

**Authors:** Ulrike Kipman, Stephan Bartholdy, Marie Weiss, Wolfgang Aichhorn, Günter Schiepek

**Affiliations:** ^1^College of Education, Institute of Educational Sciences and Research, Salzburg, Austria; ^2^Department of Psychology, University of Greifswald, Greifswald, Germany; ^3^Department of Psychology, University of Graz, Graz, Austria; ^4^Department of Psychiatry, Psychotherapy and Psychosomatics, Paracelsus Medical Private University, Salzburg, Austria; ^5^Institute of Synergetics and Psychotherapy Research, Paracelsus Medical Private University, Salzburg, Austria

**Keywords:** complex problem solving (CPS), personality disorders, behavioral characteristics, personality traits, problem solving

## Abstract

Complex problem solving (CPS) can be interpreted as the number of psychological mechanisms that allow us to reach our targets in difficult situations, that can be classified as complex, dynamic, non-transparent, interconnected, and multilayered, and also polytelic. The previous results demonstrated associations between the personality dimensions neuroticism, conscientiousness, and extraversion and problem-solving performance. However, there are no studies dealing with personality disorders in connection with CPS skills. Therefore, the current study examines a clinical sample consisting of people with personality and/or depressive disorders. As we have data for all the potential personality disorders and also data from each patient regarding to potential depression, we meet the whole range from healthy to impaired for each personality disorder and for depression. We make use of a unique operationalization: CPS was surveyed in a simulation game, making use of the microworld approach. This study was designed to investigate the hypothesis that personality traits are related to CPS performance. Results show that schizotypal, histrionic, dependent, and depressive persons are less likely to successfully solve problems, while persons having the additional behavioral characteristics of resilience, action orientation, and motivation for creation are more likely to successfully solve complex problems.

## Introduction

A *problem* arises when a person is unable to reach the desired goal. *Problem-solving* refers to the cognitive activities aimed at removing the obstacle separating the present situation from the target situation (Betsch et al., 2011). In our daily lives, we are constantly confronted with new challenges and a plethora of possibilities to address them. Accordingly, problem-solving requires the ability to identify these possibilities and select the best option in the unfamiliar situations. It is, therefore, an important competence to deal with new conditions, adapt to changing circumstances, and react flexibly to new challenges (Kipman, 2020).

Even tasks for which the sequence of choices to be taken is relatively straight-forward, such as in the process of navigating to a certain destination in a foreign city or cooperative decision-making during psychotherapy, appear as a highly diversified process, when considered in detail (Schiepek, 2009; Schiepek et al., 2016a). However, most problems we face in everyday life are not as well defined and do not necessarily have an unambiguous solution. The ability to deal with such sophisticated problems, i.e., *complex problem solving (CPS)*, is of particular relevance in everyday settings.

Funke (2001, 2003, 2012) and Dörner and Funke (2017), identified five dimensions along which complex problems can be characterized: (i) The *complexity* of the problem arises from the number of variables contributing to the problem, which in turn affect the number of possible solutions. (ii) The *connectivity* of the problem arises from the number of interconnections between these variables. (iii) The *dynamics* of the problem arise from changes in the problem variables or their interconnections over time. These changes can be a result of the person’s actions or are inherent to the problem, i.e., characteristics of the variables themselves or a result of interactions between the variables. (iv) The *non-transparency* of a problem refers to the extent to which the target situation, the variables involved, their interactions and dynamics cannot be ascertained. (v) Finally, complex problems are usually *polytelic*, i.e., they have more than one target situation.

Accordingly, CPS requires the ability to model the problem space, i.e., understand which variables are involved and how they are interconnected, the ability to handle a large number of variables at the same time, judge the relevance and success probability of possibilities, identify the interconnections between variables and the inherent dynamics thereof, judge the consequences of one’s own actions with regards to the problem space, and collect relevant knowledge to deal with non-transparency.

Tasks to measure this complex set of abilities were developed by Dörner (1980, 1986), who criticized that the measurement of general intelligence tended to use simple tasks that are not comparable with the level of complexity of real-world problems. He proposed measuring intelligent behavior in computerized environments specifically adapted to simulate the properties of sophisticated problems in everyday settings (Danner et al., 2011b). cf. Dörner et al. (1983) in research used settings referred to as *Microworlds* to assess the way participants acted under heterogeneous, dynamic, and non-transparent conditions. Participants were instructed to administrate a tiny German village by the name of Lohhausen by creating the ideal conditions for the village and its inhabitants (Hussy, 1998, p. 140–141). This microworld comprised more than 2,000 variables, guaranteeing an elevated level of complexity, which also required a high-level operationalization of CPS. However, the general validity of the performance at Lohhausen turned out to be a questionable issue, since the performance was operationalized as a factor composed of 6 main criteria, some of which were subjective assessments. Accordingly, the parameter definition for CPS performance was rather ambiguous. The reason for this ambiguity is that the vague description of the objective, i.e., to establish a respectable standard of well-being among the inhabitants—gave room for subjective interpretation (cf. Hussy, 1998, p. 146–150). Since then, the psychometric validity of the CPS performance in complex microworlds has been demonstrated by several researchers (e.g., Wittmann and Hattrup, 2004; Danner et al., 2011a).

Because of the high-translational relevance of the topic, the question arises how and which individual differences contribute to more or less efficient solving of the complex problems, such as Microworlds. Individual differences in problem-solving have been described along a cognitive dimension, i.e., the *problem-solving style*, and an emotional–motivational dimension, i.e., the *problem orientation* (D’Zurilla et al., 2011). Cognitively, problems can be solved in a *rational style*, i.e., systematically and deliberate, in an *impulsive style*, i.e., careless, hurried, and often incomplete, or in an *avoidance style via* passivity and inaction leading to procrastination (D’Zurilla et al., 2002, as cited in D’Zurilla et al., 2011). Emotionally, people with a *positive problem orientation*, see problems as an opportunity for success, i.e., a “challenge” and are confident that the problem is solvable, and that they will be able to solve it. People with a *negative problem orientation* view problems as an opportunity for failure, i.e., a “threat” and doubt their ability to solve the problem (D’Zurilla et al., 2011).

Some studies have already related basic personality traits, such as the BIG-5, to the way a person tackles complex problems. For example, it has been demonstrated that individuals who score high in conscientiousness, openness for experience, and extraversion also have higher problem-solving abilities. In contrast, individuals with higher scores in neuroticism show poor problem-solving abilities (D’Zurilla et al., 2011). McMurran et al. (2001) demonstrate that this is a result of the way in which neurotic individuals approach problems. Neuroticisms was significantly associated with an impulsive or avoidant problem-solving style, and a negative problem orientation. Vice versa, Arslan (2016) identified a positive relationship between constructive problem-solving and being extrovert, receptive, and open to new learning experiences, and also high in tolerability and accountability.

The present study seeks to extend these findings to individuals with “extreme” levels of personality traits, i.e., individuals with personality disorders, taking into consideration the way in which personality characteristics manifest in everyday situations, such as work–place situations. Following the most current diagnostic approach to personality disorders as outlined in the ICD-11, the individual accentuations of 9 disorder-relevant personality traits were taken into account, including:

(i) *Paranoid traits*, i.e., the extent of mistrust toward others.
(ii) *Schizoid traits*, i.e., the inability to express feelings and experience pleasure, resulting in fierce separation from affective contacts and also friends and social gatherings with an excessive preference for the magical worlds.
(iii) *Antisocial traits*, i.e., the extent of disregard for social obligations and callous lack of involvement in feelings for others, resulting in aggressive behavior.
(iv) *Borderline traits*, i.e., the tendency to act out impulses without regard to consequences, associated with unpredictable and erratic moods.
(v) *Histrionic traits*, i.e., the tendency to overdramatize and show a theatrical, exaggerated expression of feelings, suggestibility, egocentricity, hedonism, and a constant desire for recognition, external stimuli, and attention.
(vi) *Dependent traits*, i.e., excessive and inappropriate agreeableness (Costa and McCrae, 1986) resulting in major anxiety about separation, feelings of helplessness, and a tendency to subordinate oneself to the desires of others.
(vii) *Schizotypal traits*, i.e., extreme levels of introversion, resulting in social disengagement.
(viii) *Obsessive-compulsive (anankastic) traits*, i.e., excessive conscientiousness, involving feelings of doubt, perfectionism, and inflexibility.
(ix) *Depressive traits*, i.e., the tendency toward persistent feelings of sadness and loss of interest.

Few studies have assessed problem-solving, much less CPS, in patients with personality disorders. Previous research shows, that patients with histrionic and narcissistic personality types show an impulsive problem-solving style, whereas avoidant and dependent individuals show a negative problem orientation (McMurran et al., 2007). In addition, people who are in a depressive mood (Lyubomirsky et al., 1999), or even clinically depressed and anxious have difficulties generating effective solutions to problems (Marx et al., 1992). Accordingly, we hypothesize a negative association between high accentuations of disorder-related personality traits and CPS. The aim of the present study was to explore, which disorders were most severely affected and whether this association also manifested in work-related situations.

Action-orientated problem-solving is particularly required in areas where people are under a lot of stress, for example, in entrepreneurship, team leading in the clinical settings, or firefighting. Especially when a work-related crisis appears, action-oriented problem-solving is important, because it unites handling both novel and routine demands (Rudolph and Repenning, 2002, as cited in Rudolph et al., 2009). Rudolph et al. (2009) found that only by taking action, information cues become available. Accordingly, both CPS and everyday situations in the work-place require the ability to cope with stressful events and protect oneself from the negative effects of stress, i.e., resilience (Lee and Cranford, 2008, as cited in Wagnild and Young, 1993; Fletcher and Sarkar, 2013). Indeed, individuals with a high trait resilience are more willing to take action in problem-solving (Li and Yang, 2009, as cited in Li et al., 2013). This is consistent with previous research demonstrating that effective problem-solving abilities go along with high-psychological resilience (Garcia-Dia et al., 2013; Williamson et al., 2013; Crowther et al., 2016, as cited in Pinar et al., 2018). Pinar et al. (2018) even found that problem-solving competencies can be increased by increasing psychological resilience and self-confidence levels. Accordingly, identifying which personality disorders are most severely affected in these areas may also provide hints for psychotherapy.

## Materials and methods

### Participants

**FIGURE 1:**
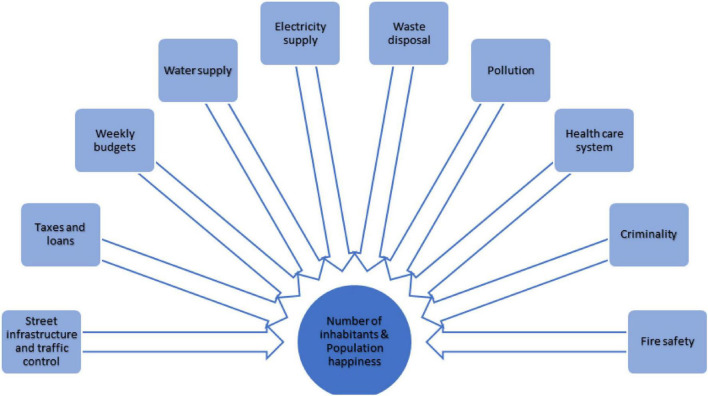
Exemplary model of some (not all) factors that influence the number of inhabitants and the general happiness of the population in Cities: Skylines (CSL). The number of related variables illustrates the complexity, connectivity, and polytely in the simulated environment.

The present study included data from *N* = 242 adults (49.1% male) with personality disorders and/or depressive disorders, with ages ranging from 17 to 48 years (mean: 26.5 years). The participants were given five assessment batteries and a set of demographic variables, which included game experience. They were also given a commercial complex problem-solving (CPS) game known as *Cities: Skylines* involving the construction and managing of a city like a mayor would with the goal of growing the city while not running out of money. Participants were patients from psychiatric and psychosomatic hospitals, who got follow-up treatment directly after leaving the hospital. The treatment took place in a panel practice for aftercare where the CPS experiment was done (see Figure 1).

### Material

#### Personality questionnaires

In order to obtain a comprehensive diagnosis and measure disordered personality traits in a continuous fashion, three personality questionnaires were used, including the PSSI, SCID-5-PD, and MMPI-II. While the PSSI scores were used in the statistical analysis, SCID-5-PD scores and MMPI-II scores were used to confirm the PSSI diagnosis. Furthermore, in order to assess the manifestation of disordered personality traits in work-related situations, we used the BIP.

The Persönlichkeits-Stil und Störungs-Inventar (PSSI; Kuhl and Kazen, 2009) is a self-report instrument that measures the comparative manifestation of the character traits. These are designed as non-pathological analogs of the personality disorders described in the psychiatric diagnostic manuals DSM-IV and ICD-10. The PSSI comprises 140 items assigned to 14 scales: PN (willful-paranoid), SZ (independent-schizoid), ST (intuitive-schizotypal), BL (impulsive-borderline), HI (agreeable-histrionic), NA (ambitious-narcissistic), SU (self-critical-avoidant), AB (loyal-dependent), ZW (conscientious-compulsive—anankastic), NT (critical-negativistic), DP (calm-depressive), SL (helpful-selfless), RH (optimistic-rhapsodic), and AS (self-assertive-antisocial). Patients rate each item on a 4-point Likert scale (from 0 to 3) and continuous scale values are calculated as the sum of the 10 item ratings belonging to a scale. Accordingly, a maximum value of 30 can be achieved for each scale. In this study, we focused on the nine traits PN, SZ, ST, BL, HI, AB, ZW, DP, and AS, as the other measured traits are not listed as personality disorders in the ICD-10 or DSM-V.

The Strukturiertes Klinisches Interview für DSM-5—Persönlichkeitsstörungen (SCID-5-PD; First et al., 2019) is a semi-structured diagnostic questionnaire that can be used to evaluate the 10 personality disorders included in the DSM-5 in clusters A, B, and C, as well as disorders in the category “not otherwise specified personality disorder.” Each DSM-5 criterion is assigned corresponding interview questions to assist the interviewer in assessing the criterion. It is possible to utilize the SCID-5-PD to categorically diagnose personality disorders (present or absent) (First et al., 2019). In addition, regulations are also included which can be used to create dimensional ratings.

The MMPI^®^ –2 (Butcher et al., 2000) is a revised and completely re-normed version of the Minnesota Multiphasic Personality Inventory (MMPI). With the help of the MMPI^®^ –2, a relatively complete picture of the personality structure can be obtained in an economical way.

The Bochumer Inventar zur berufsbezogenen Persönlichkeitsbeschreibung (BIP; Hossiep and Paschen, 2019) measures personality traits in a work-related context. A total of 210 items are assigned to 4 global dimensions including 14 subscales. These include *work orientation* (diligence, agility, and focus), *professional approach* ( performance-, creativity-, and management motivation), *social competencies* (sensitivity, social skills, sociability, teamwork, and assertiveness), and *mental constitution* (emotional stability, resilience, and self-confidence) on a continuous scale. Patients respond to each item on a 6-point Likert scale.

#### Game experience

As possible previous experience with the CPS game may affect the level of problem-solving efficiency during the test, participants were asked to rate their previous engagement with simulation-based urban development games on a 4-point Likert scale with response options running from “none” to “very much.” The same poll also featured a listing of 20 symbols from Cities: Skylines, in combination with their meanings (e.g., “no electricity”) for participants to make use of during their quest. At the end, participants were asked to rate their experience based on a 5-point scaling reaching from 1 (extremely simple) to 5 (super challenging). At last, the researcher also marked on each poll sheet, whether (a) the individual patient was able to accomplish the mission (Success, Failure, or Patient Breakup), and (b) the exact time frame of the testing session (morning, afternoon, or evening).

#### Cities: Skylines (CSL)

**FIGURE 2:**
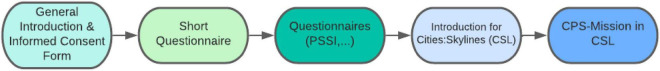
Procedure of the study.

The computer-based simulation game Cities: Skylines (Paradox Interactive, 2015a), which can be downloaded from Steam for about 30 dollars, explores the construction and management of a city and was implemented in the current study as a Microworld scenario. Much like in the successful microworld Lohhausen (Dörner et al., 1983), gamers in Cities: Skylines basically act *in lieu* of the city’s mayor, taking over all of his authority and duties. As promised in the user manual, it “offers endless sandbox play in a city that keeps offering new areas, resources, and technologies to explore, continually presenting the player with new challenges to overcome” (Paradox Interactive, 2015b, p. 4). The game fulfills the parameters of Brehmer and Dörner’s (1993) microworlds and meets the standards of complex problems according to Funke’s (2001; 2012). The examples below illustrate the way in which these features are relevant for Cities: Skylines (see Figure 2; see also de Kooter, 2015; Paradox Interactive, 2015b):

(i) *Complexity* is fulfilled because the system is made up of a variety of components including a vast series of different constructions (areas, basic resources, roads, constructions, electricity, water supplies, etc.), options (fiscal matters, budgeting, credit, traffic management, security, healthcare, and education), and parameters (population density, inhabitant satisfaction, environmental issues, and delinquency). As an example, while purchasing a wind turbine, the participant may weigh related costs, budgeted funds for the week, potential noise pollution, the way the turbine blends into the landscape vs. the rate of efficiency, along with the hardware required to connect the device to the town’s existing network, etc.
(ii) *Connectivity* is fulfilled because the parameters in the model are heavily interconnected. Each component is related to at least one other element (see Figure 2) implementing a network of correlations and interdependencies. As an example, residential zones should not be located in proximity to wind turbines, as the amount of noise pollution caused by their operation might affect the quality of life in that zone, which again might make the area less attractive and lower the property values.
(iii) *Dynamics* are fulfilled because the demands of the population are subject to autonomous change, while other variables, e.g., zoning requirements also depend in part on the actions of the participants. While the dynamics of the game cause the population and the territory of the city to grow, the whole infrastructure becomes inadequate over time and needs to be adapted. Water and electricity infrastructures, the number of schools, clinics, municipal cemeteries, etc., that used to suffice for the population then need to be expanded. Moreover, depending on its frequentation, each building or road has a certain life span until it is left abandoned and will have to be replaced.
(iv) *Non-transparency* is not featured as an essential part of the Cities: Skylines gameplay, but is instead primarily caused by its connectivity and intricacy. While playing the game, the number of variables and their interconnections make active exploration essential. Independent of the player’s actions; however, there are also very non-transparent features, such as random death waves or an (unexpectedly) higher incidence of fires in the area following the first construction of a firefighter center by the player.
(v) *Polytely* arises since the objective to increase the population of the city requires the simultaneous achievement of a large number of minor tasks, which may be conflicting (e.g., strategic allocation of bus stops for both students and employees). The situation is further complicated by unforeseen complications (e.g., water pollution causing disease spread), which force the player to abandon his/her ongoing task and give full attention to the new issue. The source of the problem must be evaluated while new strategies for potential solutions are weighed against proven approaches. For the current research, each patient was provided with identical settings, including a sizeable, completely functional city with a number of 2,600 residents, 50,000 game money points, and a general population satisfaction level of 90%. Their subsequent task was to boost the population of the cities to 5,000 residents while making sure that the residents were not poorly (as measured by an average satisfaction level of at least 75%) and the bank balance remained positive. On the contrary, the task was left unaccomplished if (a) the population of the urban areas dropped to 1,000, (b) the balance of the account dropped to 0, or (c) the maximum game time of 120 min had elapsed. Patients received the tip, that it was necessary to set priorities and focus on the mission.

Based on the task of raising the number of inhabitants of the city, a parameter of CPS performance was calculated as the average growth of the population relative to the target size of 5,000:


$$C\,P\,S=\frac{\sum p\,o\,p\,u\,l\,a\,t\,i\,o\,n\,d\,i\,f\,f\,e\,r\,e\,n\,c\,e\,s\,b\,e\,t\,w\,e\,e\,n\,t\,i\,m\,e\,p\,o\,i\,n\,t\,s}{n\,u\,m\,b\,e\,r\,o\,f\,t\,i\,m\,e\,p\,o\,i\,n\,t\,s-1}\times \frac{p\,o\,p\,u\,l\,a\,t\,i\,o\,n\,m\,a\,x\,i\,m\,u\,m}{p\,o\,p\,u\,l\,a\,t\,i\,o\,n\,g\,o\,a\,l}$$

Gamers were instructed not to modify the time settings during the game, to allow for comparable measurements across participants.

Given that the participants were patients from psychiatric and psychosomatic hospitals, many of them lacked game experience. To increase test fairness between patients with different levels of game experience, all the participants were provided with a brief introduction on how to handle a list of fundamental game features:

• placement of streets, buildings, water pumps, and wind turbines;
• positioning of roads, structures, water pipes, and turbines;
• division of zones (housing, businesses, and industries/offices zones) and the mode of bulldozing;
• structural survey of power, water lines, and waste collection;
• search for the info stats to view the requirements of the residents;

### Statistical analysis

For all the statistical analyses, SPSS version 26.0 (2020) was used.

On the basis of the ICD-11 definition, the personality traits were not analyzed categorically (as before), but dimensionally. To relate the expression of currently recognized personality disorders to performance in CPS, we used correlation analyses between CPS performance and the 9 scale scores of the PSSI (verified by the SCID and MMPI-2) and also the 4 overall dimensions of the BIP. Given the high number of resulting correlations, *p*-values could be misleading because of the multiple testing. Accordingly, we identified relevant personality traits for CPS using (i) The Bonferonni-correction of *p*-values and (ii) an effect sizes cut-off of *r* > 0.25.

In a second step, we explored, which facets of the BIP contributed to the associations with CPS performance in order to get a more fine-grained picture of possible effects.

In sum, we sought to identify the strongest predictors of CPS performance using 3 multivariate regression models with the 9 clinical traits, controlling for gender in the 2nd model and additional game experience in the 3rd model.

## Results

Table 1 lists the experience with urban planning simulation games in the current sample. About 50% of the patients rated the game as “easy” or “rather easy,” 37.5% rated it as “not easy but also not difficult” and 12.6% responded that the game was “difficult” or “very difficult.”

**TABLE 1:** Experience of the sample (*N* = 242, *N* = 210 valid answers).

	%
No experience	28.6
Some experience	57.1
Much experience	7.1
Very much experience	7.1

Correlation analyses show that CPS performance was negatively related to schizotypal (*r* = −0.46), histrionic (*r* = −0.44), and depressive (*r* = −0.46) personality accentuations. The higher the expression in any of these areas, the higher the probability of failing in CPS. Effect sizes (: = *r*) were > 0.40 for each of these traits (compare Table 2). Furthermore, CPS-performance was negatively correlated with the dependent (*r* = −0.29) and paranoid (*r* = −0.25) personality traits, but coefficients were much lower and therefore of less practical relevance as for schizotypical, histrionic, and depressive traits. Schizoid (*r* = 0.04), borderline (*r* = 0.17), anankastic (*r* = −0.05), and anti-social (*r* = −0.04) traits were not significantly associated with the CPS (see Table 3).

**TABLE 2:** Correlations of CPS and personality disorders with work-related personality manifestations as assessed with the BIP.

Variable	CPS	Paranoid	Schizoid	Schizotypal	Borderline	Histrionic	Dependent	Anancastic	Antisocial	Depressive
**Work orientation**										
Conscient.	*0.208* **	−0.067	**0.270** **	−0.066	−0.079	0.020	−0.183**	0.111	−0.220**	−0.084
Flexibility	−0.152*	−0.030	0.017	*0.230* **	−0.163*	*0.231* **	−0.057	**0.324** **	−0.175**	−0.075
Action ori.	**0.309** **	−0.151*	0.004	−0.217**	−0.051	−0.124	−0.096	−0.049	−0.005	−0.195**
**Professional orientation**										
Achievement motivation	0.174**	−0.270**	0.022	−0.013	−0.166**	0.114	−0.116	0.104	−0.122	−0.208**
Creation motivation	**0.292** **	−0.061	0.185**	−0.123	−0.100	−0.073	−0.157*	0.085	−0.041	−0.249**
Leadership motivation	*0.209* **	−0.069	0.001	−0.030	−0.056	0.056	−0.095	−0.039	−0.115	−0.337**
**Social competencies**										
Sensitivity	−0.099	−0.109	0.047	0.168**	−0.013	0.100	0.022	−0.002	−0.193**	−0.141*
Social skills	−0.152*	−0.117	0.041	**0.251** **	−0.050	*0.249* **	−0.072	−0.060	−0.260**	0.006
Sociability	−0.303**	−0.048	0.101	**0.362** **	0.087	0.167**	0.135*	−0.040	−0.201**	−0.115
Team orientation	−0.172**	−0.016	−0.171**	0.055	−0.105	0.100	0.092	−0.179**	0.061	−0.002
Assertiveness	0.091	0.167**	0.149*	−0.040	−0.132*	0.052	−0.211**	0.113	0.049	−0.078
**Psychological constitution**										
Emotional stability	0.087	0.084	0.078	−0.123	−0.296**	−0.050	−0.248**	−0.042	0.021	−0.151*
Resilience	**0.371** **	−0.080	0.036	−0.172**	−0.283**	−0.279**	−0.276**	−0.031	0.079	−0.236**
Self confidence	−0.001	0.018	0.120	0.054	−0.180**	0.151*	−0.036	0.043	−0.066	−0.125

Correlations surpassing an effect size of r = 0.25 are highlighted in bold font, italic numbers have a lower effect size but are still significant when taking only the Bonferroni Correction into account, **p < 0.01, *p < 0.05.

**TABLE 3:** Correlations between personality traits and CPS performance.

Variable	CPS	2	3	4	5	6	7	8	9	10
CPS										
Paranoid (2)	−0.254**	1								
Schizoid (3)	0.040	**0.365** **	1							
Schizotypal (4)	−0.464**	0.083	−0.079	1						
Borderline (5)	−0.171**	−0.029	−0.291**	0.193**	1					
Histrionic (6)	−0.435**	0.032	0.084	0.201**	−0.012	1				
Dependent (7)	−0.287**	−0.250**	−0.283**	0.227**	**0.431** **	−0.059	1			
Anankastic (8)	−0.049	0.046	0.123	0.160*	−0.064	**0.258** **	−0.056	1		
Antisocial (9)	−0.042	0.064	−0.027	−0.013	−0.067	−0.204**	0.137*	−0.148*	1	
Depressive (10)	−0.456**	0.224**	0.103	0.055	0.030	0.225**	0.068	0.056	0.008	1

Correlations surpassing an effect size of r = 0.25 are highlighted in bold font, **p < 0.01, *p < 0.05 but not significant when taking the Bonferroni Correction into account.

Regarding the work-related manifestations of the personality traits, CPS-performance was positively associated with the overall BIP dimensions of work orientation (*r* = 0.27), professional orientation (*r* = 0.34), and psychological constitution (*r* = 0.25), but negatively with the overall BIP dimension social competencies (*r* = −0.25). In order to explore these associations further, CPS performance and personality disorders were related to the sub-facet scores of the BIP (see Table 2).

Professional orientation was also negatively correlated with depressive traits (*r* = −0.40), the psychological constitution was negatively correlated with borderline traits (−0.38), dependent traits (−0.31), and with depressive traits (−0.26).

The results demonstrate that particularly the facets resilience, action orientation, and motivation for creation were positively correlated with successful problem-solving, while sociability and CPS were significantly negatively correlated. The higher the resilience, action orientation and motivation for creation and the lower the sociability, the better was the CPS performance. When we take Bonferroni correction into account, also conscientiousness and motivation for leadership (italic in the table) were negatively correlated with the CPS performance.

Interestingly, the associations between personality disorders and work-related personality expressions were moderate. The strongest associations arose for resilience, which was negatively associated with several personality disorders, particularly, borderline, histrionic, and dependent traits. Focusing on the traits that showed the strongest impairment in CPS, schizotypal traits were associated with high sociability (*r* = 0.36) and to a lesser extent with low-action orientation (*r* = −0.22), which in turn related to low-CPS performance. Histrionic traits were related to low resilience (*r* = −0.28), which in turn related to low-CPS performance. Depressive traits were related to low motivation for creation (*r* = −0.25), and also low-leadership motivation (*r* = −0.34) and to a lesser extent low-achievement motivation (*r* = −0.21), low-action orientation (*r* = −0.20), and low resilience (*r* = −0.24), which in turn is related to low-CPS performance.

In a combined model with all 9 personality traits (adjusted *R*^2^ = 36.7%), we confirmed that histrionic traits have the biggest negative impact on CPS performance (β = −0.351), followed by schizotypical (β = −0.312) and depressive traits (β = −0.303). Also, in the multiple regression model, dependent and paranoid traits are negatively related to CPS performance. If gender is the part of the model and held constant in a model containing the 9 traits, histrionic traits still have a significant and practical relevant impact of β′ = −0.325. (Condition Index = 24). The same holds true when also taking game experience into account (β′′ = −0.319) see Table 4.

**TABLE 4:** Combined regression model, β′: controlling for gender, β′′ controlling for gender and game experience.

Variable	β	β′	β′′
Paranoid	−0.244	−0.253	−0.236
Schizoid	0.088	0.092	0.120
Schizotypal	−0.312**	−0.297**	−0.298**
Borderline	0.023	0.020	0.056
Histrionic	−0.351**	−0.325**	−0.319**
Dependent	−0.251	−0.231	−0.205
Anankastic	0.090	0.071	0.048
Antisocial	−0.048	−0.058	−0.061
Depressive	−0.303**	−0.267	−0.254
Gender	−	−0.187	−0.144
Experience	−		−0.031

Correlations surpassing an effect size of r = 0.25 are highlighted in bold font, **p < 0.001.

(Condition Index checking for possible multicollinearity is moderate with CI = 22, 36, so multicollinearity is moderately given, βs are, therefore, interpretable, *p*-values can be slightly biased, βs with 0.3 and higher found in this model for the 3 traits have for certain a significant and practically relevant impact).

## Discussion

The present study examined the influences of personality traits on the CPS performance in a clinical sample of individuals with a range of different psychiatric diagnoses. The aim of this empirical analysis was to extend previous research on individual differences in CPS to extreme personality traits as observed in personality disorders, and also their manifestation in work-related situations. We explored, which personality dimensions were most strongly associated with impairments in the CPS.

With regards to the clinical personality dimensions (i.e., dimensionally defined personality disorders), statistical analyses revealed that schizotypal, histrionic, dependent, and depressive personality traits were associated negatively with the participants’ performances in the given CPS task (consistent with, e.g., McMurran et al., 2007). Previous findings on these relationships were, therefore, further confirmed, specifically in showing that subjects with high levels of depressiveness and anxiety seemed to have more difficulties in finding and executing effective solutions to the given complex problems (e.g., see Marx et al., 1992; Lyubomirsky et al., 1999).

Unsurprisingly, no single clinical personality structure was associated with better problem-solving performances (as compared with the non-clinical trait levels). As personality disorders are generally linked with increased levels of neuroticism, which subsequently was consistently found to negatively influence problem-solving (e.g., McMurran et al., 2001; D’Zurilla et al., 2011), this result is also consistent with the general clinical intuition. But, contrary to the previous findings (D’Zurilla et al., 2011), conscientiousness had no significant impact on CPS performance in this sample.

Further analyses gave deeper insights into relationships that were found in the first part of the data analyses. They are especially allowed to draw conclusions for the clinical patients. It was found that higher levels of resilience (consistent with, e.g., Garcia-Dia et al., 2013; Williamson et al., 2013; Crowther et al., 2016, as cited in Li and Yang, 2009; Pinar et al., 2018, as cited in Li et al., 2013), action orientation, and motivation for creation (e.g., see Eseryel et al., 2014) positively influenced the problem-solving performance as *additional behavioral characteristics*. This indicates that, even for high levels of usually negative personality traits, a person’s ability to successfully solve problems will not be impaired automatically if the person is also very resilient to the effects of negative events and highly action-oriented and motivated when facing problems. Hence, this interpretation is consistent with the conclusions of a study by Güss et al. (2017), who found that more approach-oriented individuals outperformed avoidance-oriented participants in the complex problems. In this way, these positive traits act against the negative impact of otherwise impairing personality traits or even disorders. Interestingly, sociability was found to have a negative influence on the participants’ performances, while no significant influences on social skills, team orientation, or self-confidence were found. Therefore, it seems to be more comprehensible why some of us deal easily with complex problems and can manage things forward-looking while others do not succeed in making good decisions.

## Data availability statement

The raw data supporting the conclusions of this article will be made available by the authors, without undue reservation.

## Ethics statement

Ethical review and approval was not required for the study on human participants in accordance with the local legislation and institutional requirements. The patients/participants provided their written informed consent to participate in this study.

## Author contributions

UK was the main author, did all calculations, research to and wrote the article. SB did the programming of the microworlds and all technical support. MW did the review on the introduction and discussion part. WA and GS served as a consultant. All authors contributed to the article and approved the submitted version.

## References

[B1] Arslan C. (2016). Interpersonal problem solving, self-compassion and personality traits in university students. *Educ. Res. Rev.* 11 474–481. 10.5897/ERR2015.2605

[B2] Betsch T. Funke J. PLessner H. (2011). *Denken - urteilen, entscheiden, problemlösen*. Berlin: Springer.

[B3] Brehmer B. Dörner D. (1993). Experiments with computer-simulated microworlds: Escaping both the narrow straits of the laboratory and the deep blue sea of the field study. *Comput. Hum. Behav.* 9 171–184. 10.1016/0747-5632(93)90005-D

[B4] Butcher J. N. Dahlstrom W. G. Graham J. R. Tellegen A. Kaemmer B. (2000). *Minnesota multiphasic personality inventory*^®^-2. Bern: Huber.

[B5] Costa P. T. McCrae R. R. (1986). Personality stability and its implications for clincal psychology. *Clin. Psychol. Rev.* 6 407–423. 10.1016/0272-7358(86)90029-2

[B6] Crowther S. Hunter B. McAra-Couper J. Warren L. Gilkison A. Hunter M. (2016). Sustainability and resilience in midwifery: A discussion paper. *Midwifery* 40 40–48. 10.1016/j.midw.2016.06.005 27428097

[B7] Dörner D. (1980). On the difficulties people have in dealing with complexity. *Simul. Games* 11, 87–106.

[B8] Dörner D. (1986). Diagnostik der operativen intelligenz. *Diagnostica* 32, 290–308.

[B9] D’Zurilla T. J. Maydeu-Olivares A. Gallardo-Pujol D. (2011). Predicting social problem solving using personality traits. *Pers. Individ. Differ.* 50 142–147. 10.1016/j.paid.2010.09.015

[B10] D’Zurilla T. J. Nezu A. M. Maydeu-Olivares A. (2002). *The Social Problem-Solving Inventory-Revised (SPSI-R): Technical Manual.* North Tonawanda, NY: Multi-Health Systems, Inc.

[B11] Danner D. Hagemann D. Holt D. V. Hager M. Schankin A. Wüstenberg S. (2011a). Measuring performance in dynamic decision making. *J. Individ. Differ.* 32 225–233. 10.1027/1614-0001/a000055

[B12] Danner D. Hagemann D. Schankin A. Hager M. Funke J. (2011b). Beyond IQ: A latent state-trait analysis of general intelligence, dynamic decision making, and implicit learning. *Intelligence* 39 323–334. 10.1016/j.intell.2011.06.004

[B13] de Kooter S. (2015). *Cities Skylines Guide: Beginner Tips and Tricks Guide.* Available Online at: https://www.gameplayinside.com/strategy/cities-skylines/cities-skylines-guide-beginner-tips-and-tricks-guide/.

[B14] Dörner D. Funke J. (2017). Complex problem solving: What it is and what it is not. *Front. Psychol.* 8:1153. 10.3389/fpsyg.2017.01153 28744242 PMC5504467

[B15] Dörner D. Kreuzig H. W. Reither F. Stäudel T. (1983). Lohhausen: Vom Umgang mit Unbestimmtheit und Komplexität. *Soziologische Rev.* 8:93.

[B16] Eseryel D. Law V. Ifenthaler D. Ge X. Miller R. (2014). An investigation of the interrelationships between motivation, engagement, and complex problem solving in game-based learning. *Educ. Technol. Soc.* 17 42–53.

[B17] First M. Williams J. Smith B. Spitzer R. (2019). *SCID-5-PD.* Göttingen: Hogrefe.

[B18] Fletcher D. Sarkar M. (2013). A review of psychological resilience. *Eur. Psychol.* 18 12–23. 10.1027/1016-9040/a000124

[B19] Funke J. (2001). Dynamic systems as tools for analysing human judgement. *Think. Reason.* 7 69–89. 10.1080/13546780042000046 23334335

[B20] Funke J. (2003). *Problemlösendes Denken.* Stuttgart: Kohlhammer.

[B21] Funke J. (2012). “Complex Problem Solving,” in *Encyclopedia of the Sciences of Learning*, ed. Seel N. M. (Boston, MA: Springer US), 682–685. 10.1007/978-1-4419-1428-6_685

[B22] Garcia-Dia M. J. DiNapoli J. M. Garcia-Ona L. Jakubowski R. O’Flaherty D. (2013). Concept analysis: Resilience. *Arch. Psychiatr. Nurs.* 27 264–270. 10.1016/j.apnu.2013.07.003 24238005

[B23] Güss C. D. Burger M. L. Dörner D. (2017). The role of motivation in complex problem solving. *Front. Psychol.* 8:851. 10.3389/fpsyg.2017.00851 28588545 PMC5440455

[B24] Hossiep R. Paschen M. (2019). *Bochumer Inventar zur berufsbezogenen Persönlichkeitsbeschreibung*. Göttingern: Hogrefe.

[B25] Hussy W. (1998). *Denken und Problemlösen.* Stuttgart: Kohlhammer.

[B26] Kipman U. (2020). *Komplexes problemlösen*. Wiesbaden: Springer.

[B27] Kuhl J. Kazen M. (2009). *Persönlichkeits-Stil-und Störungs-Inventar*. Göttingen: Hogrefe.

[B28] Lee H. H. Cranford J. A. (2008). Does resilience moderate the associations between parental problem drinking and adolescents’ internalizing and externalizing behaviors?: A study of Korean adolescents. *Drug Alcohol Depend.* 96 213–221. 10.1016/j.drugalcdep.2008.03.007 18440164

[B29] Li M. H. Yang Y. (2009). Determinants of problem solving, social support-seeking, and avoidance: A path analytic model. *Int. J. Stress Manag.* 16 155–176. 10.1037/a0016844

[B30] Li M. H. Eschenauer R. Yang Y. (2013). Influence of efficacy and resilience on problem solving in the United States, Taiwan, and China. *Multicult. Couns. Dev.* 41 144–157. 10.1002/j.2161-1912.2013.00033.x

[B31] Lyubomirsky S. Tucker K. L. Caldwell N. D. (1999). Why ruminators are poor problem solvers: Clues from the phenomenology of dysphoric rumination. *J. Pers. Soc. Psychol.* 77 1041–1060. 10.1037/0022-3514.77.5.1041 10573879

[B32] Marx E. M. Williams J. M. Claridge G. C. (1992). Depression and social problem solving. *J. Abnorm. Psychol.* 101 78–86. 10.1037/0021-843X.101.1.78 1537977

[B33] McMurran M. Duggan C. Christopher G. Huband N. (2007). The relationships between personality disorders and social problem solving in adults. *Pers. Individ. Differ.* 42 145–155. 10.1016/j.paid.2006.07.002

[B34] McMurran M. Egan V. Blair M. Richardson C. (2001). The relationship between social problem-solving and personality in mentally disordered offenders. *Pers. Individ. Differ.* 30 517–524. 10.1016/S0191-8869(00)00050-7

[B35] Paradox Interactive (2015a). *Cities: Skylines.* Stockholm, SE: Paradox Interactive.

[B36] Paradox Interactive (2015b). *Cities: Skylines: User Manual.* Stockholm, SE: Paradox Interactive.

[B37] Pinar S. E. Yildirim G. Sayin N. (2018). Investigating the psychological resilience, self-confidence and problemsolving skills of midwife candidates. *Nurse Educ. Today* 64 144–149. 10.1016/j.nedt.2018.02.014 29482050

[B38] Rudolph J. W. Repenning N. P. (2002). Disaster dynamics: Understanding the role of quantity in organizational collapse. *Adm. Sci. Q.* 47 1–30. 10.2307/3094889

[B39] Rudolph J. W. Morrison J. B. Carroll J. S. (2009). The dynamics of action-oriented problem solving: Linking interpretation and choice. *Acad. Manag. Rev.* 34 733–756. 10.5465/AMR.2009.44886170

[B40] Schiepek G. (2009). Complexity and nonlinear dynamics in psychotherapy. *Eur. Rev.* 17 331–356. 10.1017/S1062798709000763

[B41] Schiepek G. Stöger-Schmidinger B. Aichhorn W. Schöller H. Aas B. (2016a). Systemic case formulation, individualized process monitoring, and state dynamics in a case of dissociative identity disorder. *Front. Psychol. Clin. Settings* 7:1545. 10.3389/fpsyg.2016.01545 27812338 PMC5072376

[B42] Wagnild G. M. Young H. M. (1993). Development and psychometric evaluation of the resilience scale. *J. Nurs. Meas.* 1 165–178. 7850498

[B43] Williamson G. R. Health V. Proctor-Childs T. (2013). Vocation, friendship and resilience: A study exploring nursing student and staff views on retention and attrition. *Open J. Nurs.* 7 149–156. 10.2174/1874434601307010149 24167537 PMC3807580

[B44] Wittmann W. W. Hattrup K. (2004). The relationship between performance in dynamic systems and intelligence. *Syst. Res. Behav. Sci.* 21 393–409. 10.1002/sres.653

